# Retrospective study of preoperative chemoradiotherapy with capecitabine versus capecitabine plus oxaliplatin for locally advanced rectal cancer

**DOI:** 10.1038/s41598-020-69573-z

**Published:** 2020-07-27

**Authors:** Xiao-Hui Yang, Kai-Guo Li, Jun-Bao Wei, Chun-Hua Wu, Shi-Xiong Liang, Xian-Wei Mo, Jian-Si Chen, Wei-Zhong Tang, Song Qu

**Affiliations:** 1 Department of Radiation Oncology, Guangxi Medical University Cancer Hospital, Cancer Institute of Guangxi Zhuang Autonomous Region, Nanning, 530021 Guangxi People’s Republic of China; 2 Guangxi Clinical Research Centre for Colorectal Cancer, Guangxi Medical University Cancer Hospital, Nanning, 530021 Guangxi People’s Republic of China

**Keywords:** Cancer therapy, Gastrointestinal cancer, Gastrointestinal cancer

## Abstract

This study aimed to evaluate whether the addition of oxaliplatin to a neoadjuvant chemoradiotherapy (CRT) regimen could improve survival benefit in locally advanced rectal cancer (LARC) patients. We retrospectively analysed 73 LARC patients (cT2-4 and/or cN1-2) who received preoperative CRT with capecitabine followed by surgery (arm A, 43 patients) or capecitabine plus oxaliplatin followed by surgery (arm B, 30 patients). The main endpoints of the study were pathologic complete response (pCR) rate, overall survival (OS) and disease-free survival (DFS). The secondary endpoints included the sphincter preservation rate and safety. The pCR for arms A and B were 28% and 17% (*P* = 0.267). In arms A and B, the mean OS was 84.287 months (95% CI 68.413–100.160) and 106.333 months (95% CI 99.281–113.386) (*P* = 0.185); the mean DFS was 72.812 months (95% CI 56.271–89.353) and 95.073 months (95% CI 83.392–106.754) (*P* = 0.310); and the sphincter preservation rates were 72% and 67%, respectively (*P* = 0.619). The incidence of grade 3 toxicity was much higher in arm B than in arm A (57% vs. 21%, *P* = 0.002). Adding oxaliplatin to a preoperative CRT regimen for LARC did not improve the survival benefits of patients or increase toxicity.

## Introduction

At present, rectal cancer is one of the main causes of cancer death worldwide. According to the latest cancer statistics in America^1^, the mortality rate of colorectal cancer is the second highest. Colorectal cancer has the third highest incidence of cancer and the fifth highest mortality, according to the published report of cancer epidemiology in China, 2015^2^. The high morbidity and mortality of rectal cancer has posed a serious threat to people's health.

Radiation, chemotherapy and surgery have played crucial roles in the multimodality therapy strategy of rectal cancer in the past few decades^3^. Many researchers have preferred to treat colorectal cancer with a preoperative chemoradiotherapy (CRT) regimen^4,5^. The results of a randomized clinical trial^6^ in Germany showed that preoperative CRT was better than postoperative CRT in terms of local control and adverse event occurrence for rectal cancer (T3–4 or node positive). For the preoperative CRT of rectal cancer, a number of concurrent chemotherapy regimens are available.

In the clinical setting, capecitabine (Xeloda) is commonly used in concurrent chemoradiation. Capecitabine is a novel tablet form of fluoropyrimidine that can more easily transform into 5-fluorouracil (5-FU) by thymidine phosphorylase (TP) in cancer cells. Many previous retrospective studies^7–10^ and a randomized study^11^ have suggested that compared with intravenously administered fluorouracil/leucovorin, oral capecitabine achieved a higher tumour reaction rate. In addition, capecitabine is more convenient and has good tolerance.

With the development of drugs for treating locally advanced rectal cancer (LARC) patients, some scholars have proposed the use of capecitabine plus oxaliplatin (XELOX) for treatment. Oxaliplatin is a third-generation platinum derivative whose target is tumour cell DNA, and it mainly inhibits DNA synthesis by causing intrastrand cross-linking in DNA and producing cytotoxicity^12^. Its antitumour spectrum is more extensive than that of other platinum drugs, especially in the treatment of colorectal cancer, showing good therapeutic characteristics, which is a major step forward in the treatment of colorectal cancer. Oxaliplatin is an ideal radiosensitizer. Recently, the radiotherapy sensitization of oxaliplatin has been found in in vivo and in vitro studies^13^. Randomized clinical tests^14,15^ have shown that oxaliplatin combined with FU/leucovorin is superior to FU/leucovorin monotherapy in terms of antitumour activity in metastatic colorectal carcinoma.

The therapeutic effect and adverse reactions of oxaliplatin added to the preoperative CRT regimen for rectal cancer have not been very clear, and there are few comparative studies on this topic. Therefore, the purpose of this study was to compare the clinical outcomes of two preoperative CRT regimens, capecitabine alone and XELOX, in LARC.

## Results

### Patient characteristics

A total of 73 patients who met our inclusion criteria were included in this study (43 in arm A and 30 in arm B); the median ages were 57.0 [30–74] and 56.7 [29–78]. Both arms were predominantly male (70% in arm A and 73% in arm B). Regarding the TNM classification, the patients were more often T3-4 node positive. The two groups were well balanced for age, sex, T and N stage and so on. Table 1 lists the distributions of patient characteristics in the two groups.

**Table 1:** Baseline characteristics of patients.

Characteristic	Radiotherapy plus capecitabine (n = 43)		Radiotherapy plus XELOX (n = 30)		*P*
	No.	%	No.	%	
**Median age, years**	57.0		56.5		0.618
Range	30–74		29–78		
**Sex**					0.741
Male	30	70	22	73	
Female	13	30	8	27	
**Location from anal verge, cm**					0.746
< 5 cm	26	60	17	57	
5–10 cm	17	40	13	43	
**Clinical T category**					0.134
T2	0	0	3	10	
T3	22	51	14	47	
T4	21	49	13	43	
**Clinical N category**					0.077
N0	9	21	12	40	
N1-2	34	79	18	60	
**Tumour differentiation**	43		30		0.086
Well differentiated	2	5	4	13	
Moderately differentiated	39	90	21	70	
Poorly differentiated	2	5	5	17	

The patient characteristics were similar for both treatment arms. More patients in arm A than in arm B had tumours within 5 cm from the anal edge (60% vs. 57%), but the difference was not significant (*P* = 0.746). Similarly, there was no significant difference in the differentiation level of tumour cells between the two groups (*P* = 0.086).

### Surgery and pathology findings

Table 2 displays the results of this analysis. Although the capecitabine group tended to have a higher rate of sphincter preservation, the difference between the two groups (67% in the XELOX group and 72% in the capecitabine group) was not statistically significant (*P* = 0.619). In arm A, pCR was confirmed in 12 patients (28%). In arm B, pCR was confirmed in 5 patients (17%). However, this difference was not statistically significant (*P* = 0.267).

**Table 2:** Pathological features of the surgical specimen.

End Point	Radiotherapy plus capecitabine (n = 43)		Radiotherapy plus XELOX (n = 30)		*P*
	No.	%	No.	%	
pCR	12	28	5	17	0.267
Sphincter-saving surgery rates	31	72	20	67	0.619
TRG*					0.603
4: complete regression	12	28	5	17	
3: > 50% of tumour mass	7	16	7	23	
2: > 25–50% of tumour mass	21	49	17	57	
1: < 25% of tumour mass	3	7	1	3	
0: no regression	0	0	0	0	
**Resection status**					1.000
R0	39	90.7	28	93.3	
R1	4	9.3	2	6.7	

After neoadjuvant CRT, there was no difference between the two arms in tumour regression (*P* = 0.603). The complete resection (R0) rates were also similar in the two groups (90.7% vs. 93.3%). There was no statistically significant difference in the R0 resection rate (*P* = 1.000). Macroscopic residual tumours (R2 resection) were not recorded in the postoperative pathologic reports of all patients in either group.

### Acute toxicity and postoperative complications

Table 3 summarizes the adverse events of the two groups according to the National Cancer Institute Common Toxicity Criteria grade. There were no deaths related to toxicity or treatment intolerance in the two groups, despite patients experiencing some adverse events during the study. Although the incidence of grade 4 toxicity was similar in both groups (*P* = 0.639), grade 3 toxicities were more frequent in the XELOX group (21% and 57%, respectively, *P* = 0.002). This difference was mainly due to grade 3 radioactivity cystitis and haematological toxicity. There were also differences in the incidence of individual adverse events between the two groups. The capecitabine group had more grade 3 radiation dermatitis (5% vs. 3%) and grade 1/2 leukopenia (53% vs. 47%) events than the XELOX group, while the XELOX group had more grade 3 diarrhoea (20% vs. 12%) and grade 1/2 anaemia (17% vs. 12%) events. As expected, the incidence of grade 1/2 peripheral neuropathy was statistically significant in arms A and B (0 vs. 13%, *P* = 0.025).

**Table 3:** Adverse reaction.

Toxicity by grade	Radiotherapy plus capecitabine (n = 43)		Radiotherapy plus XELOX (n = 30)		*P*
	No.	%	No.	%	
4 All toxicity	3	7	1	3	0.639
**3**					
All toxicity	9	21	17	57	0.002
Radioactivity cystitis	0	0	4	13	0.025
Diarrhoea	5	12	6	20	0.325
Hematologic	4	9	9	30	0.05
Radiation dermatitis	2	5	1	3	1.000
**2-1**					
Anaemia	5	12	5	17	0.538
Digestive system	24	56	19	63	0.521
Radiation dermatitis	20	47	13	43	0.788
Peripheral neuropathy	0	0	4	13	0.025
Leukopenia	23	53	14	47	0.566
Thrombocytopenia	4	9	4	3	0.872

Table 4 shows the results of the postoperative complications comparison between the two groups. There was no significant difference in the incidence of postoperative complications between the two groups (28% vs. 37%, *P* = 0.428). 14 percent of patients in group B and 2 percent in group A developed varying degrees of anastomotic inflammation after radical operation. Moreover, the difference was statistically significant (*P* = 0.017).

**Table 4:** Postoperative complications.

Complications	Radiotherapy plus capecitabine (n = 43)		Radiotherapy plus XELOX (n = 30)		*P*
	No.	%	No.	%	
All complications	12	28	11	37	0.428
Anastomotic inflammation	1	2	6	14	0.017
Bowel obstruction	4	9	4	13	0.872
Bleed	0	0	2	7	0.166

### Survival

**Figure 1:**
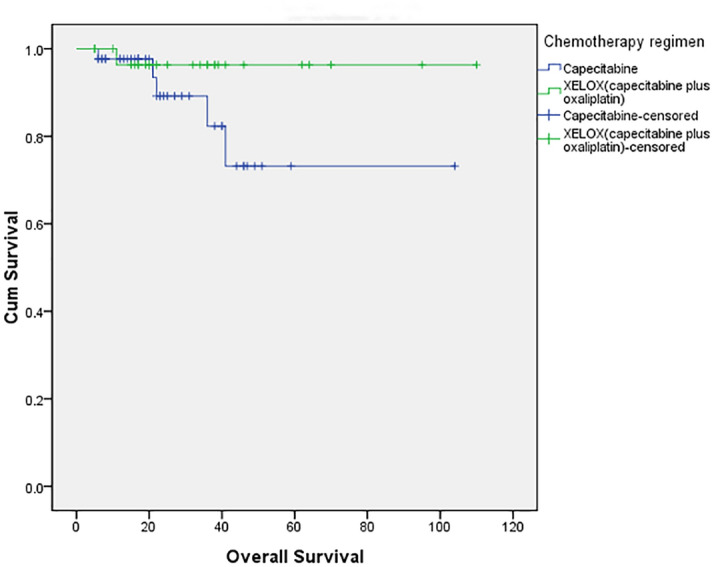
Overall survival curves for the capecitabine and XELOX groups among patients with locally advanced rectal cancer. The OS rates were not significantly different between the two groups (*P* = 0.185).

**Figure 2:**
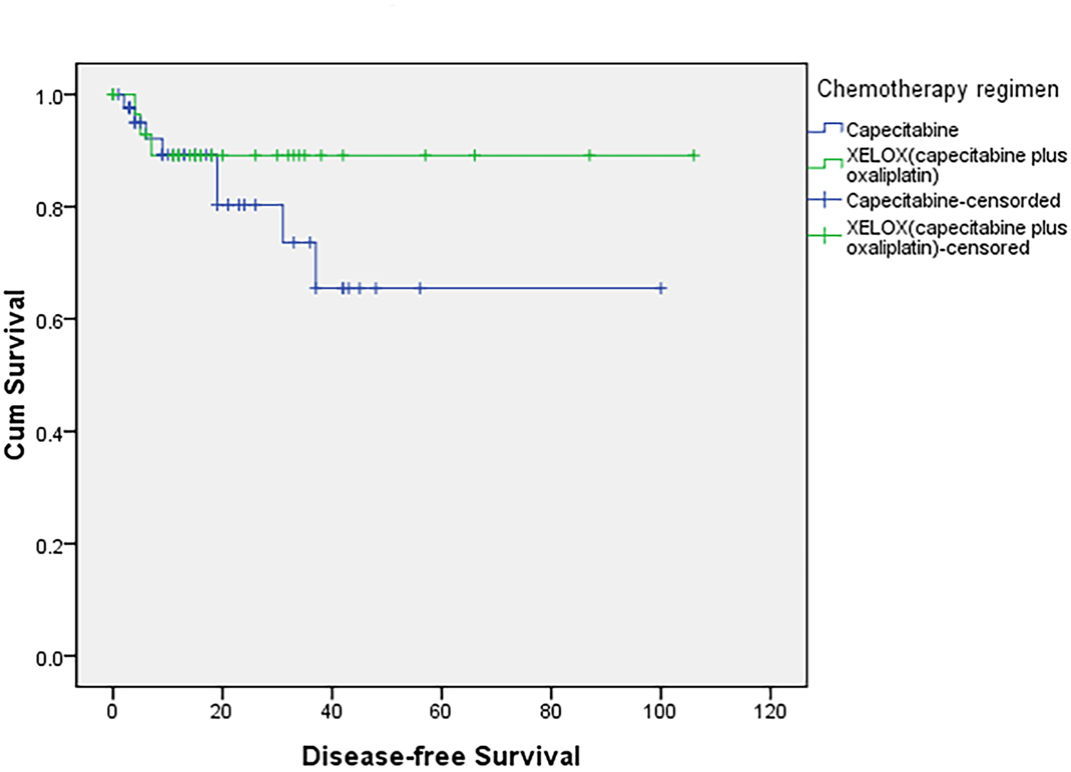
Disease-free survival curves for the capecitabine and XELOX groups among patients with locally advanced rectal cancer. The DFS rates were not significantly different between the two groups (*P* = 0.310).

The median follow-up time for 73 patients was 22 months (range 5–110 months). The mean OS was 84.287 months (95% CI 68.413–100.160) in the capecitabine group vs. 106.333 months (95% CI 99.281–113.386) in the XELOX group (*P* = 0.185). No statistically significant differences were noted in the OS between the two groups (Fig. 1). The mean DFS was 72.812 months (95% CI, 56.271 to 89.353) in the single drug capecitabine group and 95.073 months (95% CI 83.392–106.754) in the XELOX group (*P* = 0.310). No statistically significant differences between the study groups were observed (Fig. 2).

## Discussion

Neoadjuvant CRT has been the standard treatment for patients with LARC. After neoadjuvant CRT for rectal cancer, a large number of previously unresectable tumours can be treated with radical resection. Fluoropyrimidine-based neoadjuvant chemoradiation for LARC can significantly reduce the local recurrence rate, but the distant metastasis rate is still high^16^. To further improve the curative effect, researchers have increasingly focused on exploring new chemotherapy drugs. Oxaliplatin has attracted the attention of an increasing number of researchers due to its remarkable efficacy in adjuvant and palliative treatments of colorectal cancer, and a series of clinical studies have been carried out. However, the application of oxaliplatin in preoperative CRT remains controversial.

Large international randomized phase III clinical trials^17–21^ demonstrated that the addition of oxaliplatin to fluoropyrimidine-based CRT regimens did not improve the therapeutic effect for rectal cancer patients. Instead, the combination of drugs increased acute toxicity, limiting its efficacy. For these reasons, some investigators did not recommend the application of oxaliplatin in neoadjuvant therapy for LARC.

Similar to those of most previous studies, our results showed that oxaliplatin did not significantly improve the rate of pCR or survival benefit but significantly increased adverse reactions. In the XELOX group, the incidence of grade 3 toxicities was significantly higher than that in the capecitabine group (*P* = 0.002). This difference was mainly due to grade 3 radioactivity cystitis and haematological toxicity. Furthermore, a major side reaction of oxaliplatin was neurotoxicity. In this study, there was a significant difference in the incidence of neurotoxicity between the two groups (*P* = 0.025). Peripheral neuropathy is probably the most common adverse event associated with the clinical use of oxaliplatin. Peripheral neuropathy is cumulative and dose limiting^22^. In recent years, oxaliplatin has been widely used in the treatment of many types of malignancies, such as gastric cancer, oesophageal cancer, and ovarian cancer. Even though oxaliplatin exerts a wide range of antitumour activities in a wide variety of cancers, antineoplastic therapy with oxaliplatin may be limited by several toxic responses. The frequently reported adverse events for oxaliplatin are gastrointestinal toxicity, haematological toxicity, hypersensitivity and neurological toxicity.

We wonder whether oxaliplatin plays a role as a radiosensitizer in the preoperative CRT regimen based on fluorouracil. However, the preliminary results of the German CAO/ARO/AIO randomized phase 3 trial^3^ were contrary to the above clinical study conclusions. The results showed that oxaliplatin-containing combinations could increase the pCR rate, and adverse reactions were tolerable. The final results of this study were published in 2015^23^. The study suggested that oxaliplatin combined with 5-FU as neoadjuvant CRT plus adjuvant chemotherapy was safe and feasible and significantly improved DFS. Oxaliplatin was recommended to be included in preoperative CRT for rectal cancer, and the results were obviously distinct from those of the above four clinical trials.

The role of oxaliplatin in neoadjuvant therapy for LARC is still controversial, and the clinical benefits are limited, which may be due to the following reasons. First, different accumulated doses and application styles of oxaliplatin and fluoropyrimidines in clinical trials at each centre may have contributed to the differences in results^17,18^. Second, we conjecture that the dose of capecitabine is sufficient to maximize radiosensitivity in preoperative CRT regimens containing a single drug, while the dose of capecitabine in preoperative CRT regimens containing a combination of drugs correspondingly decreases, resulting in a low pCR rate. Of course, we can also consider capecitabine as the best radiosensitizer, which maximizes the local tumour response in combination with radiotherapy, and the additional radiosensitizers have little room for further enhancement^17^. Third, it is also possible that the optimization of radiotherapy techniques and/or higher radiation doses resulted in the difference in outcomes^24,25^.

In addition to capecitabine and oxaliplatin, to achieve a higher pCR rate and better survival outcomes as much as possible, a series of clinical trials in which new chemotherapy agents were applied to preoperative treatment in LARC have been conducted internationally in recent years. Encouraging results were obtained. These drugs include irinotecan and bevacizumab.

Irinotecan is an anticancer drug that inhibits topoisomerase and is widely used in the treatment of non-small cell lung cancer and metastatic colorectal cancer. Experiments in vitro and in vivo showed that irinotecan might act as a radiosensitizer by inhibiting various DNA repair machineries after DNA damage^26^. The results of the study by Takeo et al. and Nakamura T et al. showed that the preoperative regimen of irinotecan/S-1 and radiotherapy achieved higher response rates and excellent long-term survival, with acceptable adverse effects in patients with rectal cancer^27,28^. Both domestic and international studies have shown that irinotecan in combination with capecitabine-based CRT is feasible for LARC^29,30^.

Bevacizumab is an anti-VEGF humanized monoclonal antibody that is widely used as an antiangiogenic drug in the clinic. Bevacizumab can achieve radiosensitization by inhibiting angiogenesis^31^. A series of clinical trials have been conducted to investigate its role in neoadjuvant CRT of the rectum. Chinese scholars have evaluated the safety and efficacy of neoadjuvant XELOX combined with bevacizumab plus radiotherapy for LARC. Research has shown that the addition of bevacizumab to neoadjuvant CRT resulted in a satisfactory pCR rate and 3-year survival but might also increase the risk of anastomotic leakage^32^.

After detailed analysis of the data, there could be several reasons for these negative results. First, the small sample size inevitably caused bias, so the sample size should be further expanded for in-depth research. It is possible that with an increased sample size, a significant difference may have been detected. Second, the current follow-up time is not long enough compared with the development of the disease. Third, our research is based on a single-centre retrospective study, and the findings should be corroborated by multicentre prospective studies. We will further address these limitations and perform more extensive validation studies in the future.

Based on these research results, the routine use of oxaliplatin on the basis of capecitabine is still not recommended in preoperative CRT for LARC.

## Patients and methods

This study included 73 patients between November 2009 and March 2019 who received a long course of CRT followed by radical surgery in our centre.

The data were retrospectively analysed from the patients’ medical records, including details on patient information, clinical stage, pathological classification, radiation, chemotherapy, adverse reactions, recurrence, metastasis and survival.

### Eligibility criteria

We screened all rectal cancer patients who received preoperative CRT at Guangxi Medical University Cancer Hospital.

The inclusion criteria were as follows: (1) patients were diagnosed with rectal cancer by digital rectal examination (DRE) and endoscopic biopsy simultaneously; (2) the tumour edge from the anal margin within 12 cm was found by colonoscopy; (3) no metastasis occurred before treatment; (4) patients had not received prior radiotherapy in the pelvic area or prior cytotoxic chemotherapy; (5) the preoperative chemotherapy regimen was either capecitabine or XELOX; and (6) patients had at least one imaging evaluation during treatment.

The exclusion criteria were as follows: (1) no radical surgery was performed after the completion of preoperative CRT; (2) patients also received chemotherapy other than capecitabine or XELOX during CRT treatment; (3) patients did not complete all the planned CRT regimens; (4) patients had received any systemic antitumour therapy before the diagnosis of rectal cancer.

### Treatment

Radiotherapy: Over a period of approximately five weeks, from Monday to Friday, a total of 50–50.4 Gy high-energy photons were transferred in 25–28 fractions. A three- or four-field technique was used to irradiate the narrow pelvic volume, containing mesorecta and rectal lymph nodes, but not including external iliac lymph nodes.

Chemotherapy: during radiotherapy in arm A, capecitabine was delivered at a dose of 1,650 mg/m^2^ bid for 14 days every 21 days. In arm B, patients received the XELOX regimen composed of a 2-h venous injection of oxaliplatin 225 mg/m^2^ on the first day of the week and oral capecitabine 825 mg/m^2^ twice daily for 2 weeks. Capecitabine was first administered on the first night and last administered on the 15th morning.

Surgery: four weeks after the completion of preoperative CRT, the patients underwent computed tomography (CT) scans of the chest, abdomen and pelvis to evaluate whether there were signs of metastasis. If there was no evidence of metastasis, total mesorectal excision (TME) was performed six to eight weeks after the end of radiotherapy.

### Histopathologic assessment of the response to CRT

Specimens from the surgery were sent to the centre’s pathology department. Tumour or fibrotic areas were identified and described macroscopically after the excised specimen was processed. Pathology experts conducted standardized pathological examinations of the postoperative specimens. According to the scoring scale established by Dworak et al.^33^, a semiquantitative evaluation of residual tumour masses was conducted as follows: level 0, no reaction; level 1, minimal reaction (a few tumour cells were eliminated and a large number of residual tumour cells remained); level 2, moderate reaction (single or small clusters of cancer cells remained); level 3, good reaction (most of the tumour cells were eliminated); and level 4, total reaction (no live tumour cells were found). Pathologic complete response (pCR) was defined as no evidence of residual tumour cells in the operative specimens both at the primary site and at resected lymph nodes.

### Data management and statistics

The main endpoints of the study were pCR, overall survival (OS) and disease-free survival (DFS). OS was calculated from the date of diagnosis until death from any cause or loss to follow-up. DFS was defined as the time between tumour resection and the first incidence of disease progression or death. The Kaplan–Meier method was used to calculate OS and DFS, and the log-rank test was used for comparisons between groups. If there was no remarkable difference between groups, then other factors, such as sphincter preservation rate and incidence of adverse events, would help to select a preferred treatment. The secondary end points included the rate of sphincter preservation and safety. Where appropriate, we used the chi-square test or Fisher's exact test to compare categorical variables. All reported *P* values are two-sided. *P* < 0.05 was considered statistically significant. SPSS software (ver.17.0; IBM Corp., Chicago, IL, USA) was used for statistical analysis.

### Ethics approval and consent to participate

This study was approved by the Ethics Committee of Guangxi Medical University Cancer Hospital [approval no. LW2019005]. This study was conducted in accordance with the Declaration of Helsinki, and we ensured confidentiality of the patient data. Because it was a retrospective study, some patients died before the study, so we were unable to obtain informed consent. All patients’ information was anonymous. We obtained informed consent exemptions approved by the ethics committee.

## Data Availability

The datasets used and/or analysed during the current study are available from the corresponding author on reasonable request.
